# Loss of neutrophil polarization in colon carcinoma liver metastases of mice with an inducible, liver-specific IGF-I deficiency

**DOI:** 10.18632/oncotarget.24593

**Published:** 2018-02-28

**Authors:** Roni F. Rayes, Simon Milette, Maria Celia Fernandez, Boram Ham, Ni Wang, France Bourdeau, Stephanie Perrino, Shoshana Yakar, Pnina Brodt

**Affiliations:** ^1^ Departments of Surgery, McGill University and the McGill University Health Centre, Montréal, QC, Canada; ^2^ Department of Basic Science and Craniofacial Biology, New York University College of Dentistry, New York, NY, USA; ^3^ Department of Medicine, McGill University and the McGill University Health Centre, Montréal, QC, Canada; ^4^ Department of Oncology, McGill University and the McGill University Health Centre, Montréal, QC, Canada

**Keywords:** IGF-I, colorectal carcinoma, liver metastasis, neutrophil polarization, tumor microenvironment

## Abstract

The growth of cancer metastases in the liver depends on a permissive interaction with the hepatic microenvironment and neutrophils can contribute to this interaction, either positively or negatively, depending on their phenotype. Here we investigated the role of IGF-I in the control of the tumor microenvironment in the liver, using mice with a conditional, liver-specific, IGF-I deficiency (iLID) induced by a single tamoxifen injection. In mice that had a sustained (3 weeks) IGF-I deficiency prior to the intrasplenic/portal inoculation of colon carcinoma MC-38 cells, we observed an increase in neutrophil accumulation in the liver relative to controls. However, unlike controls, these neutrophils did not acquire the (anti-inflammatory) tumor-promoting phenotype, as evidenced by retention of high ICAM-1 expression and nitric oxide production and low CXCR4, CCL5, and VEGF expression and arginase production, all characteristic of the (pro-inflammatory) phenotype. This coincided with an increase in apoptotic tumor cells and reduced metastasis. Neutrophils isolated from these mice also had reduced IGF-IR expression levels. These changes were not observed in iLID mice with a short-term (2 days) IGF-I depletion, despite a 70% reduction in their circulating IGF-I levels, indicating that a sustained IGF-I deficiency was necessary to alter the neutrophil phenotype. Similar results were obtained with the highly metastatic Lewis lung carcinoma subline H-59 cells and in mice injected with an IGF-Trap that blocks IGF-IR signaling by reducing ligand bioavailability. Our results implicate the IGF axis in neutrophil polarization and the induction of a pro-metastatic microenvironment in the liver.

## INTRODUCTION

The tumor microenvironment is a complex network of immune and non-immune host cells that communicate with each other and with the invading cancer cells through soluble mediators and cell-cell contact to regulate tumor cell growth. A better understanding of the molecular mechanisms underlying this multifaceted communication could lead to better control of tumor progression.

Cells of the innate immune system can promote or inhibit tumor growth, depending on their phenotype. Macrophages and neutrophilic granulocytes (neutrophils) in particular, have an inherent plasticity and their phenotypes can change within a spectrum of activation states in response to chemokines and cytokines in the tumor microenvironment [1, 2]. Thus, tumor-associated macrophages (TAM) were shown to polarize from a tumoricidal M1 to a tumor-promoting M2 phenotype [3] (and reviewed in [4, 5]) and a similar N1-N2 polarization spectrum was demonstrated for neutrophils [6] (and reviewed in [7, 8]).

The type 1 insulin-like growth factor (IGF-I) axis has been implicated in several aspects of cancer progression (reviewed in [9]). Binding of IGF-I to its cell surface receptor IGF-IR results in receptor autophosphorylation and the activation of downstream effectors of the PI3K/AKT and MAPK signaling pathways that lead to cell growth, proliferation and survival [10, 11]. IGF-IR overexpression and/or autocrine activation were documented in various human malignancies (reviewed in [9, 12]). The IGF axis has also been implicated in paracrine as well as autocrine regulation of innate and acquired immunity [13]. IGF-IR is expressed on different immune cell types including T- and B-lymphocytes [14], peripheral blood mononuclear cells [15] and NK-cells [16] and these cells are therefore susceptible to regulation by IGF-I. In addition, IGF-IR has been implicated in NF-κB-mediated transcriptional regulation of inflammatory cytokines and vascular endothelial cell adhesion receptors, such as intercellular adhesion molecule-1 (ICAM-1) [17]. The IGF axis may therefore play a role in maintenance of immune homeostasis, as well as in the induction of the acute inflammatory response that accompanies the early stages of liver metastasis [12, 18]. Recently, peripheral blood CD11b^+^GR1^+^ cells were shown to express high levels of IGF-IR. IGF-IR silencing in these cells resulted in a decrease in the number of circulating neutrophils [19]. Moreover, in a mouse model of liver IGF-I deficiency (LID), congenital loss of IGF-I was associated with a decrease in the number of myeloid derived progenitor cells [20]. Additionally, IGF-IR silencing by a small interfering RNA was shown to induce the production of the pro-inflammatory cytokines TNF-α and INF-γ and consequently, alter host immunity in a mouse model of breast cancer [21]. Thus, IGF-I appears to play an active role in the regulation of immune cell function.

The liver is the main source of endocrine IGF-I production. Previously, we have shown that in mice with a congenital liver IGF-I deficiency (LID), the growth of colon carcinoma liver metastases was markedly reduced. [22]. To better understand the effects of liver-derived IGF-I on the growth of hepatic metastases, we used here mice with a conditional, tamoxifen (TX)-inducible liver IGF-I deficiency (the iLID mice), where liver IGF-I depletion can be induced in a temporal manner. Recently we reported that in mice with a prolonged (3 weeks - iLID^3W^) but not short-term (2 days- iLID^2D^) liver IGF-I depletion, hepatic stellate cells were not activated in response to tumor cell invasion and could not be rescued from apoptosis in the presence of TNFα. In these mice, a significant reduction in the number and size of colon cancer colonies in the liver was also documented [23]. Neutrophils provide a first line of defense against liver-infiltrating tumor cells. We therefore analyzed, here, how a sustained IGF-I depletion affects their recruitment to the liver following tumor cell entry and assessed the role of IGF-I in regulating their state of polarization.

## RESULTS

### A sustained reduction in liver IGF-I production alters neutrophil recruitment into the liver in response to metastatic cancer cells

**Figure 1:**
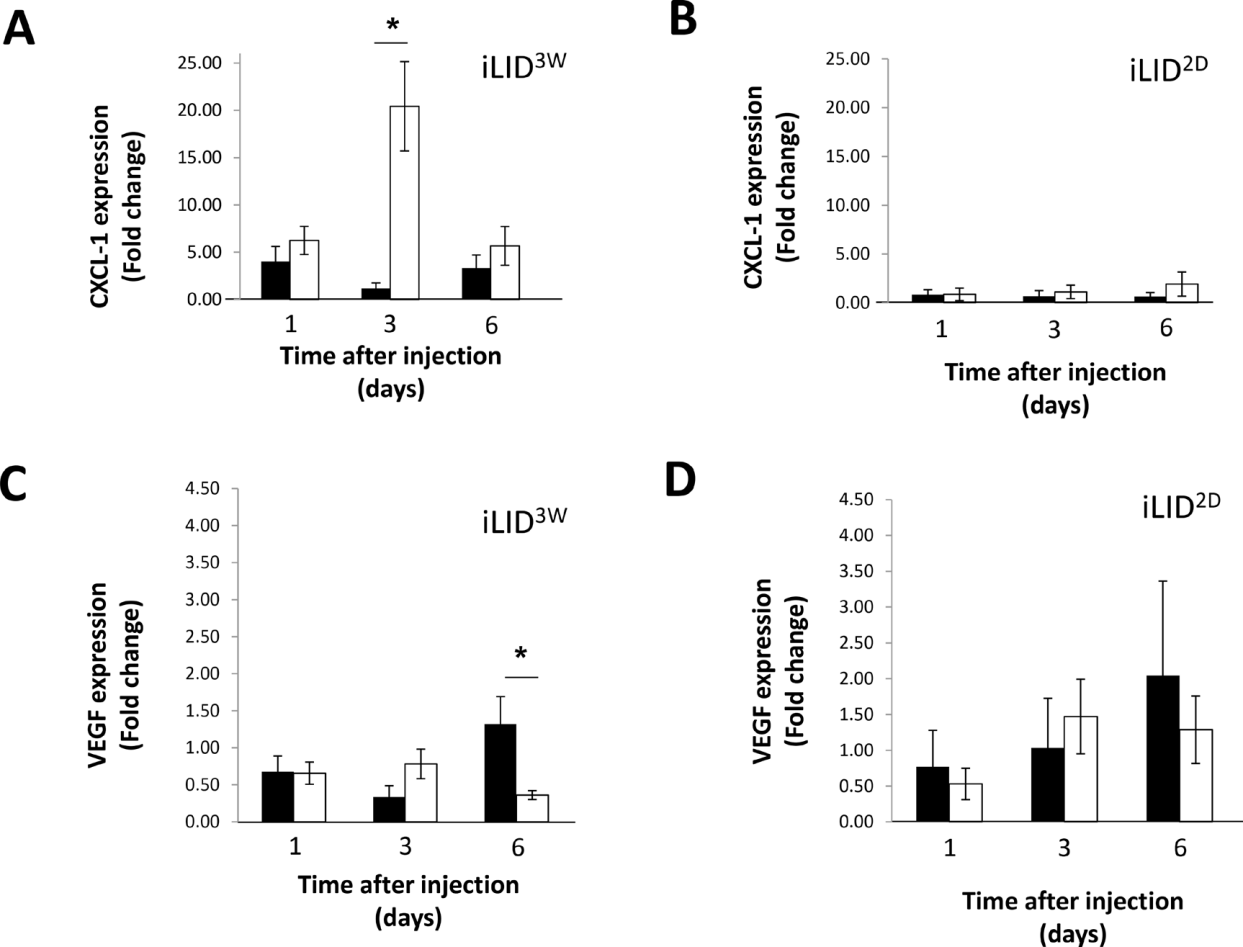
Altered chemokine/cytokine expression in tumor-injected mice with a sustained IGF-I deficiency iLID mice were injected i.p. with TX or sunflower seed oil (vehicle) 3 weeks (**A**, **C**) or 2 days (**B**, **D**) prior to the injection of 2.5 × 10^5^ MC-38-GFP cells via the intrasplenic/portal route. Mice were sacrificed 1, 3 or 6 days post tumor injection and liver fragments used for RNA extraction and analysis. Shown are expression levels of CXCL-1 (A, B) and VEGF (C, D) in TX- (white bar) and vehicle- (black bar)-injected mice, each normalized to GAPDH. The data are expressed as fold change (±SE) relative to non-injected mice that were assigned a value of 1. They are based on 3 mice per group per time point; ^*^p < 0.05.

The entry of metastatic cancer cells into the liver initiates an inflammatory cascade that leads to the recruitment and activation of innate immune response cells. Previously we have shown that in mice with a sustained liver IGF-I deficiency, induced in iLID mice by a TX injection, 3 weeks prior to the intraspelnic/portal injection of colon carcinoma MC-38 cells, liver colonization was reduced. An impaired hepatic stellate cell activation was also documented in these mice [23]. To begin to elucidate the effect of a long-term reduction in circulating IGF-I levels on the immune microenvironment of metastatic cells in the liver, we first measured the expression of a range of inflammatory/immune response indicators such as chemokines, cytokines and vascular cell adhesion molecules in the livers of iLID mice injected with GFP-tagged MC-38 cells, 1–6 days earlier. In mice with a short-term (2 days, ILID^2D^) or sustained (3 weeks, ILID^3W^) IGF-I depletion, a marked reduction (up to 90%) in circulating IGF-I levels, as compared to control mice was seen (Supplementary Table 1 and Figure 1). In all mice, we observed an increase in CXCL-1 expression levels within 24 hr of tumor injection and this persisted for at least 6 days (Figure 1A, 1B). However, while in livers of control (vehicle-treated) mice or iLID^2D^ mice -(Figure 1B), these increases ranged from 1.5 - 5-fold relative to normal (tumor-free) controls, the increase in iLID^3W^ was of a greater magnitude and was as high as 20-fold relative to the respective controls, 3 days post tumor cell inoculation (Figure 1A). Differences were also observed in VEGF expression levels in these mice. Namely, while in control, vehicle-treated and iLID^2D^ mice an increase in VEGF mRNA relative to normal levels was evident by 6 days post tumor injection (Figure 1D), these levels did not change in iLID^3W^ mice and were ∼3-fold lower than in their respective, vehicle-treated controls (Figure 1C). mRNA expression levels for other inflammatory indicators including IL-1β, IL-18, CXCL-2, E-selectin, P-selectin, VCAM-1 and ICAM-1 did not significantly differ in iLID^3W^, iLID^2D^ and the respective control mice (Supplementary Figure 2).

### Increased accumulation of Ly6G^+^ cells in iLID mice with a sustained IGF-I deficiency

**Figure 2:**
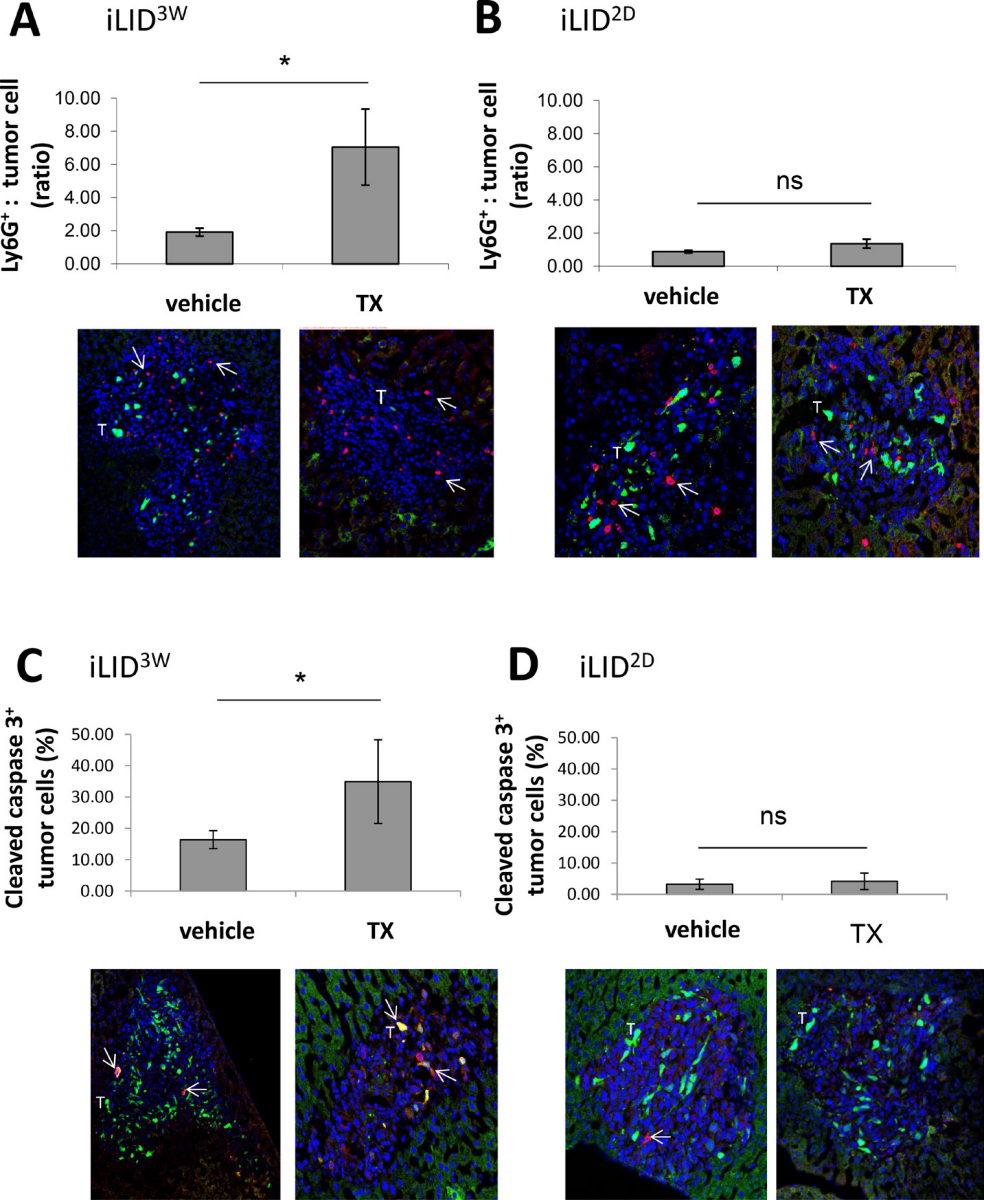
Increased neutrophil accumulation and tumor cell apoptosis in IGF-I deficient mice iLID mice were injected i.p. with TX or vehicle, 3 weeks (**A**, **C**) or 2 days (**B**, **D**) prior to the injection of 2.5 × 10^5^ MC-38-GFP cells via the intrasplenic/portal route. Mice were sacrificed 6 days post tumor injection and livers first perfused with PFA and then excised for subsequent immunohistochemical analysis. Shown in the bar graphs (A, B) are the mean numbers (±SE) of Ly6G^+^ cells counted in tumor infiltrated areas of the liver (top). They are based on 12 random fields (×10 objective), counted per condition and expressed as a ratio to the number of GFP^+^ MC-38 cells in each field. Representative images are shown on the bottom with DAPI in blue, GFP^+^ MC-38 cells (T) in green and Ly6G^+^ cells in red (arrows). Shown in (C, D) are results of quantification of MC-38 cells that expressed cleaved caspase 3, counted in 15 random fields (×10 objective) per condition and expressed as % (±SE) of the total number of tumor cells per field. Representative images are shown at the bottom with DAPI in blue, GFP^+^ MC-38 cells (T) in green and cleaved caspase 3-positive cells in red (arrow). *n* = 3. ns = not significant, ^*^p < 0.05.

The marked increased in CXCL-1 expression was indicative of a change in the immune microenvironment of the livers in iLID^3W^ mice. To identify changes in the composition of the innate immune cell infiltrate that were associated with hepatic metastases in these mice, IHC was performed on frozen liver sections derived from tumor-injected mice, 3 and 6 days post tumor injection. Consistent with our observation of increased CXCL-1 expression levels, we observed on day 6, a ∼4-fold increase in the accumulation of Ly6G^+^ cells, as compared to controls, in areas of the liver infiltrated by tumor cells (Figure 2A) and this was not observed in iLID^2D^ mice (Figure 2B). Concomitant with this increase, we found in these mice a ∼2-fold increase in the proportion of apoptotic MC-38-GFP^+^ cells, as determined by cleaved caspase 3 levels detectable by IHC (Figure 2C) and this was also not observed in the iLID^2D^ mice (Figure 2D). The reason for the slight increase in apoptosis in control (vehicle-treated) iLID^3W^ mice, relative to control iLID^2D^ mice (*p* > 0.05) is not presently clear and may have been due to the difference in the age of these mice (iLID^3W^ mice were at least 3 weeks older) [24]. Interestingly, the total number of CD11b^+^Ly6G^+^ cells in the livers of iLID^3W^ mice, as determined by flow cytometry, was not significantly altered (Supplementary Figure 3), indicating a selective enrichment of these cells in the tumor microenvironment.

### Neutrophils in mice with a sustained IGF-I depletion do not polarize to the (tumor promoting) phenotype

**Figure 3:**
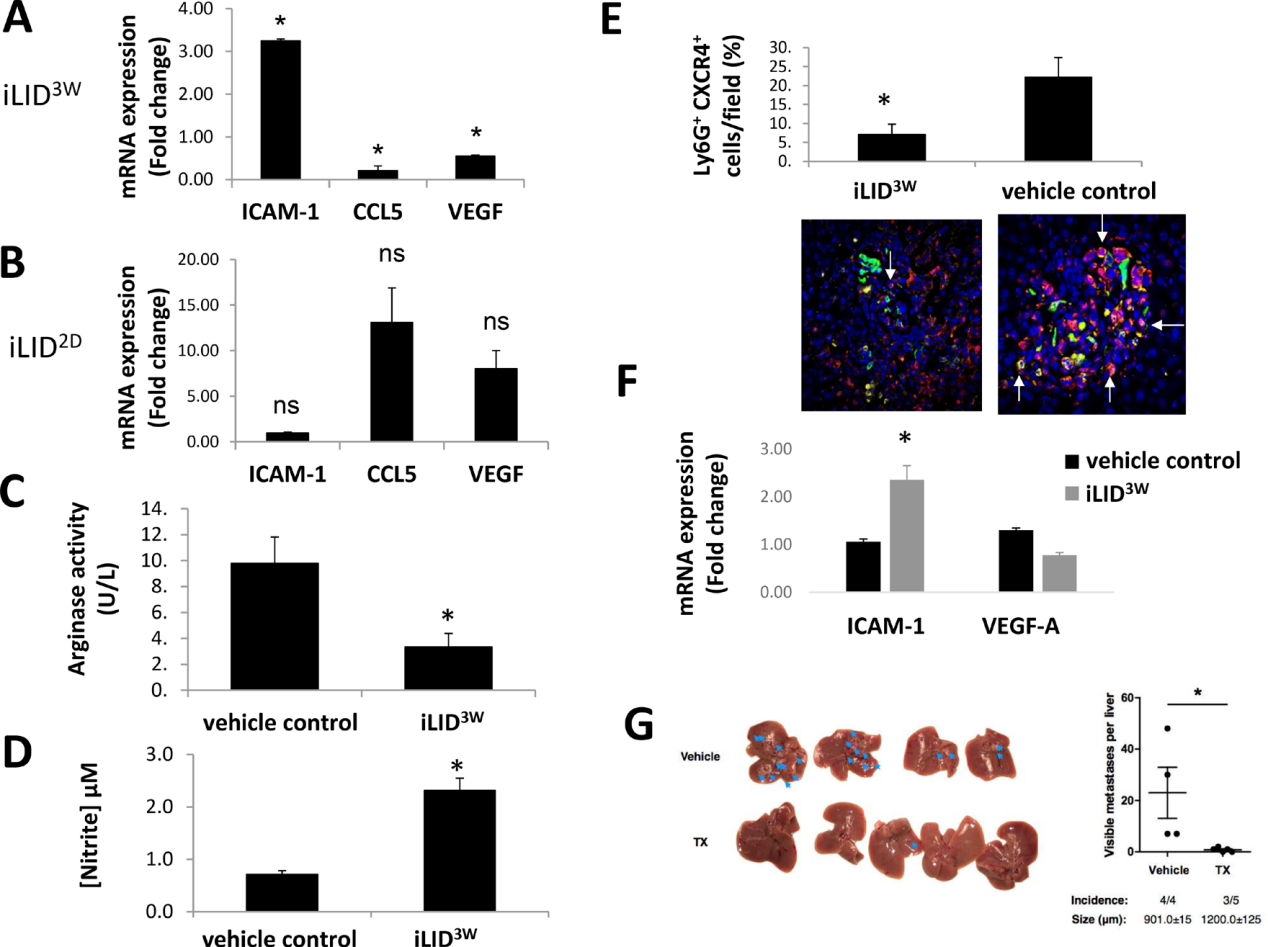
Reduced neutrophil polarization in mice with a sustained IGF-I deficiency Mice were injected as described in the legend to Figure 2 and sacrificed 6 days later. (**A–E**). Immune cells were collected and CD11b^+^Ly6G^+^ cells isolated using FACS sorting. Shown in (A) and (B) are results of qPCR performed on RNA extracted from CD11b^+^Ly6G^+^ cells isolated from iLID^3W^ (A) or iLID^2D^ (B) mice. They are based on 3 separate experiments (7–10 mice per group per experiment) and normalized to GAPDH and expressed as a ratio to the respective vehicle treated control mice. Shown in (C) are results of the arginase assay performed on total lysates of isolated CD11b^+^Ly6G^+^ cells. They are based on 3 separate experiments (7–10 mice per group per experiment) and expressed as means (±SE). Shown in (D) are nitrate levels measured in conditioned media of CD11b^+^Ly6G^+^ cells isolated from the indicated mice and incubated at 37° C overnight. They are based on 3 separate experiments (7–10 mice per group per experiment) and expressed as means (±SE). Shown in (E) are results of immunohistochemistry performed on cryostat sections derived from MC-38 cells-injected mice. Representative images of double stained cells (white arrow) with antibodies to Ly6G (in yellow) and CXCR4 (in red) around tumor cells (green) are shown on the bottom with DAPI (in blue). Results in the bar graph (top) are based on quantification performed on 8–10 sections for 3 different mice per group (*n* = 3 mice) and expressed as percent (±SE) of Ly6G^+^ cells that also stained positively for CXCR4^+^. (**F**) iLID mice were injected i.p. with TX or vehicle, 3 weeks prior to the injection of 2.5 × 10^5^ MC-38 cells via the intrasplenic/portal route. Mice were sacrificed 6 days post tumor injection and hepatic immune cells isolated. CD11b^+^Ly6G^+^ cells were sorted by FACS and RNA extracted for RT-qPCR analysis. Data in the bar graph are expressed as mean fold change (±SE) relative to vehicle control, based on 3 separate experiments (5–7 mice per group). (**G**) iLID mice received a single i.p. injection of 0.3 mg TX or sunflower seed oil (vehicle) 3 weeks prior to the injection of 10^5^ H-59 cells via the intrasplenic/portal route. Mice were sacrificed 14 days following tumor cell injection and metastases on the surfaces of the livers enumerated. Shown in the graph are the numbers of metastases seen on the individual livers in each group. Horizontal bars denote medians. The number of mice in each group that developed hepatic metastases is indicated on the bottom of each column. The average size of the metastases (±SD) is shown below each column. *n* = 4–5; ns = not significant, ^*^p < 0.05. Representative livers from each group are shown on the left. Metastases are indicated by arrows.

To better characterize the population of neutrophils that accumulated in the livers of tumor bearing mice, CD11b^+^Ly6G^+^ cells were isolated using fluorescence-activated cell sorting, RNA was extracted and the expression of pro-inflammatory neutrophil markers [8] was analyzed by qPCR. When CD11b^+^Ly6G^+^ cells isolated from livers of MC-38 - inoculated iLID^3W^ mice were analyzed, we found that their ICAM-1 expression levels were 3-fold higher, while their CCL5 and VEGF expression levels were 4.5 and ∼2-fold lower, respectively, than in the same cells isolated from the control mice (Figure 3A). This indicated that in the iLID^3W^ mice, in contrast to controls, tumor associated neutrophils (TAN) did not lose the expression of markers characteristic of the pro-inflammatory (N1) phenotype (high ICAM-1 and low CCL5 and VEGF expression [8]). These phenotypic differences were not observed in CD11b^+^Ly6G^+^ cells isolated from iLID^2D^ mice where, in fact, a trend (*p* > 0.05) toward increased CCL5 and VEGF and decreased ICAM-1 expression levels relative to the controls was observed (Figure 3B), consistent with increased polarization of these cells to a tumor-promoting (N2) phenotype (Figure 3B). The variability in the expression levels seen in the latter cells may reflect the spectrum of polarization states characteristic of tumor-associated inflammatory cells [6], and this, in turn, may be affected by the proximity of the neutrophils to the metastases. The difference between neutrophils isolated from iLID^3W^ and iLID^2D^ mice was confirmed functionally, when arginase activity levels in these cells were measured, revealing that neutrophils isolated from iLID^3W^ mice had a 3-fold reduction in this activity (Figure 3C), while their nitric oxide level was 3-fold higher (Figure 3D) than in the controls. This suggested that iLID^3W^ neutrophils did not acquire the functional properties of tumor-promoting neutrophils. Consistent with these results, an IHC analysis of Ly6G^+^ cells in liver cryostat sections derived from tumor-inoculated mice showed that approximately 20% of the neutrophils in tumor-infiltrated areas in control mice expressed the N2 marker CXCR4, as compared to only 5% in iLID^3W^ mice (Figure 3E). A similar difference in the phenotype of CD11b^+^Ly6G^+^ neutrophils was also observed following the injection of highly metastatic lung carcinoma H-59 cells into iLID^3W^ mice. These neutrophils expressed a 2.2-fold higher ICAM-1 level (*p* < 0.005) and a 1.7-fold lower VEGF-A level (*p* = 0.11) relative to neutrophils of control, wild type mice injected with TX (Figure 3F). Moreover, similarly to our findings in mice injected with colon carcinoma MC-38 cells ([23] and see also here Supplementary Figure 4), these mice also had reduced numbers of liver metastases, as compared to the controls (Figure 3G). Together, these results indicated that in mice with a sustained liver IGF-I depletion, the ability of neutrophils to polarize to a tumor-promoting phenotype was impaired and this effect was not tumor specific.

Of note, we have observed that MC-38 and H-59 cells were insensitive to stimulation by estrogen (data not shown). This, and the data shown in Figures 3F and Supplementary Figure 4 suggest that TX injections did not have a direct effect on the tumor cells *in vivo*. However, we cannot entirely rule out the possibility that within 2 days of injection, TX metabolites had indirect effects on the tumor microenvironment.

### IGF-IR expression is markedly reduced in CD11b^+^Ly6G^+^ cells isolated from iLID^3W^ mice

**Figure 4:**
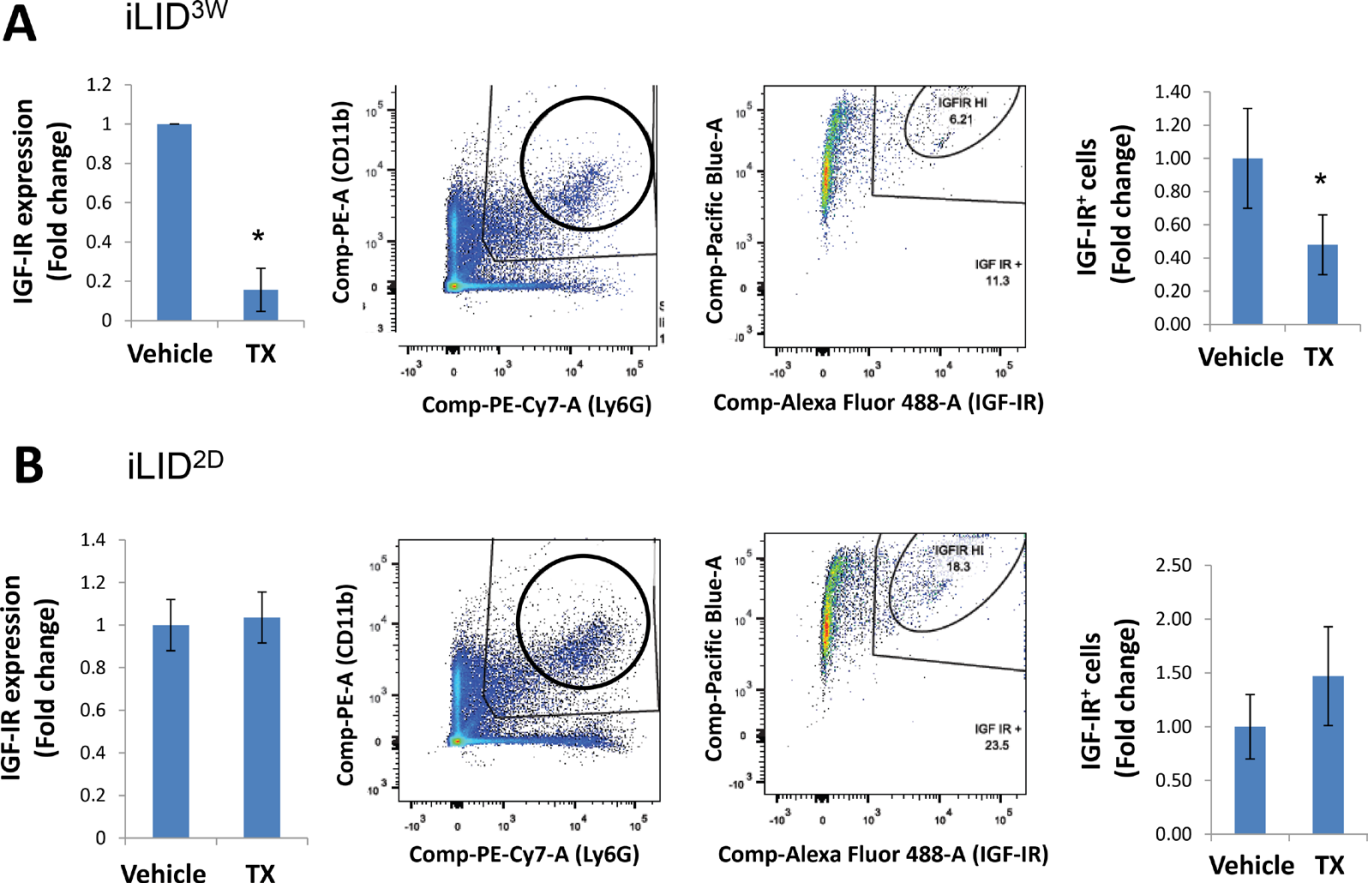
A sustained IGF-I depletion reduces IGF-IR expression levels in neutrophils ILID mice were injected with TX or vehicle 3 weeks (**A**) or 2 days (**B**) prior to the injection of 5 × 10^4^ MC-38 cells via the intrasplenic/portal route. Mice were sacrificed 6 days later and CD11b^+^Ly6G^+^ cells isolated and processed for qPCR (left) or flow cytometry (middle and right panels) using an anti IGF-IR antibody (diluted 1:100) and an Alexa Fluor 488 secondary antibody (diluted 1:200) for the latter. Shown are IGF-IR mRNA levels normalized to GAPDH (left panels), flow cytometry profiles (middle panels) and the percent IGF-IR^+^ neutrophils (right panels) expressed as means (±SE) of three experiments per condition (and 3 mice per experiment) relative to the respective, vehicle-injected mice that were assigned a value of 1. ns = not significant, ^*^p < 0.05.

The finding that neutrophils from iLID^3W^ mice were phenotypically distinct from those exposed to reduced IGF-I levels for only 2 days prompted us to ask whether this may be due to differences in their IGF-IR expression levels. IGF-IR expression levels on CD11b^+^Ly6G^+^ cells isolated from these mice were analyzed by qPCR and flow cytometry. In neutrophils isolated from iLID^3W^ mice, we found a ∼5-fold decrease in IGF-IR mRNA and a ∼2-fold decrease in IGF-IR protein levels, as compared to controls (Figure 4A), and this was not observed in neutrophils from iLID^2D^ mice (Figure 4B). In comparison to the latter, IGF-IR expression levels on neutrophils from iLID^3W^ mice were reduced by >65%.

### IGF-I can directly induce changes in the neutrophil phenotype

**Figure 5:**
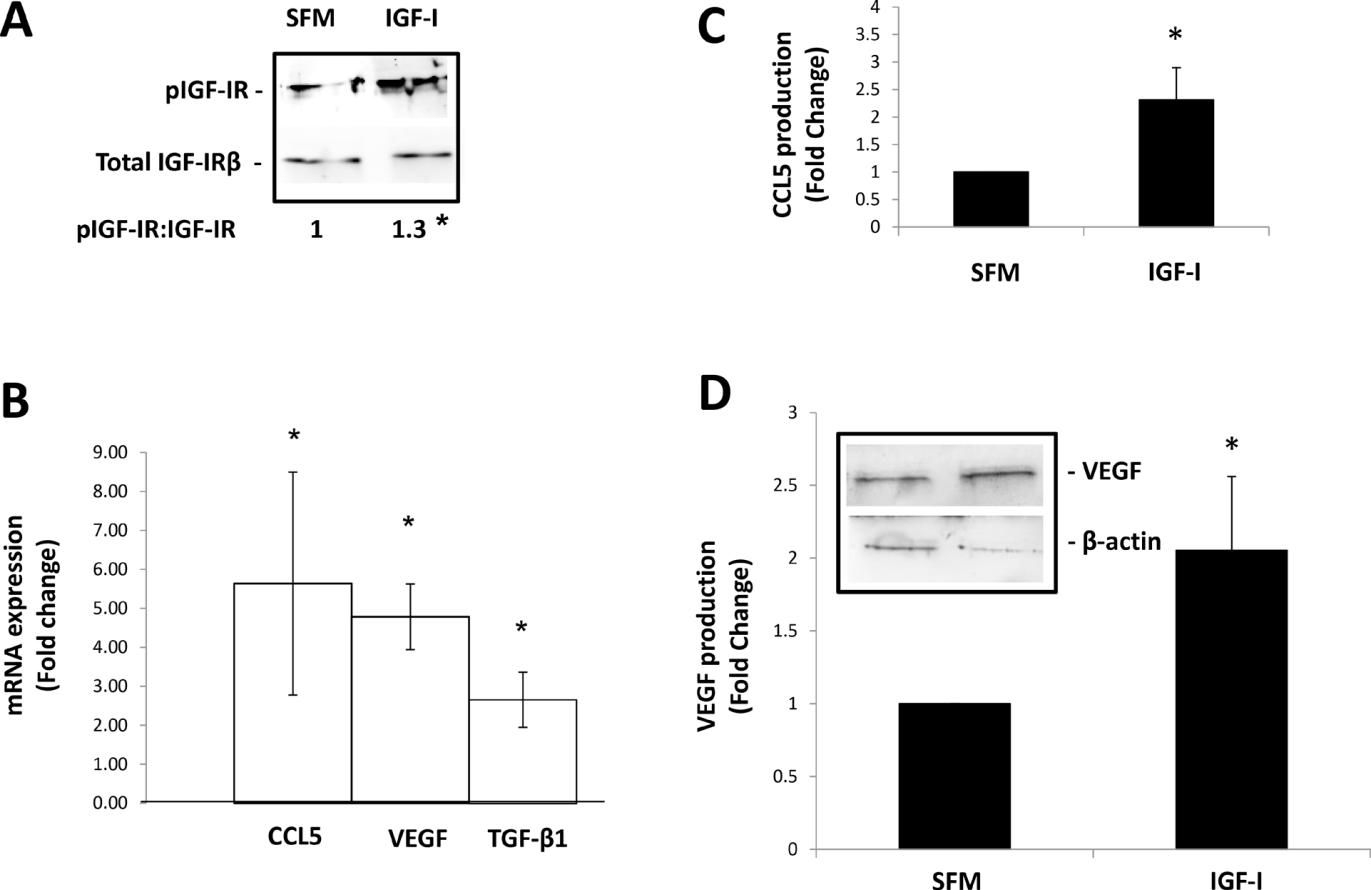
IGF-I can directly induce the expression of mRNA transcripts that define the tumor-promoting phenotype of neutrophils CD11b^+^Ly6G^+^ cells were isolate from the bone marrow of C57BL/6 mice using FACS sorting and cultured *in vitro* in serum free media (SFM) containing (or not) 10 ng/ml IGF-I for either 5 minutes prior to cell lysis for Western blotting (**A**), 3–4 hours prior to RNA extraction and analysis by qPCR (**B**) or 7–8 hours prior to collection of conditioned media for analysis by ELISA or Western blotting (**C**, **D**). Shown in (A) is a representative immunoblot performed on cell lysate proteins (3 mice/experiment/condition) and (on the bottom) the ratios of pIGFIR:IGFIR based on 3 different experiments and normalized to β-actin that was used as a loading control. Shown in (B) are mean expression values of the indicated transcripts normalized to GAPDH and expressed as fold change relative to non-treated cells that were assigned a value of 1 (*n* = 3). Shown in (C) are results of ELISA and in (D) results of immunoblotting, both performed on conditioned media collected from cultured neutrophils. They are expressed as means of fold increase (±SE) relative to unstimulated cells that were assigned a value of 1. A representative immunoblot is shown in the inset (D). The values shown in the bar graph were normalized to β-actin levels in the total cell lysates obtained from the neutrophils that were used as source of conditioned media (*n* = 3). ^*^p < 0.05.

The above results implicated IGF-I and the IGF-IR in neutrophil polarization. To determine whether IGF-I could directly induce the phenotypic changes associated with the N1-N2 transition, CD11b^+^Ly6G^+^ cells were isolated from mouse bone marrow and stimulated *in vitro* with 10 ng/ml IGF-I for 3–4 hours, prior to RNA extraction for analysis by qPCR or for 6–8 hours prior to cell lysis for protein analysis by Western blotting or ELISA. We observed a small (∼1.3-fold) but reproducible (*p* < 0.05) increase in IGF-IR phosphorylation following IGF-I stimulation for 10 minutes (Figure 5A). This was followed 3–4 hr later by a 4.5-fold increase in CCL5 and VEGF expression levels (Figure 5B) and a corresponding 2-fold increase in CCL5 and VEGF protein production levels, as measured 3–4 hr later (Figure 5C and 5D). These results showed that IGF-I could directly activate the production of N2-associated proteins in the isolated neutrophils. In addition, we also observed in IGF-I -stimulated neutrophils a ∼2-fold increase in the expression of TGF-β1 (Figure 5B) - a growth factor previously implicated in neutrophil polarization. Because most antibodies to phospho-IGF-IR also recognize the activated form of the insulin receptor (IR), we cannot entirely rule out the possibility that the IR was also activated in these cells. However, given the 100-fold lower affinity of IGF-I for IR (as compared to IGF-IR) [25], the transcriptional activation of CCL5 and VEGF in these neutrophils was most likely mediated downstream of IGF-IR.

**Figure 6:**
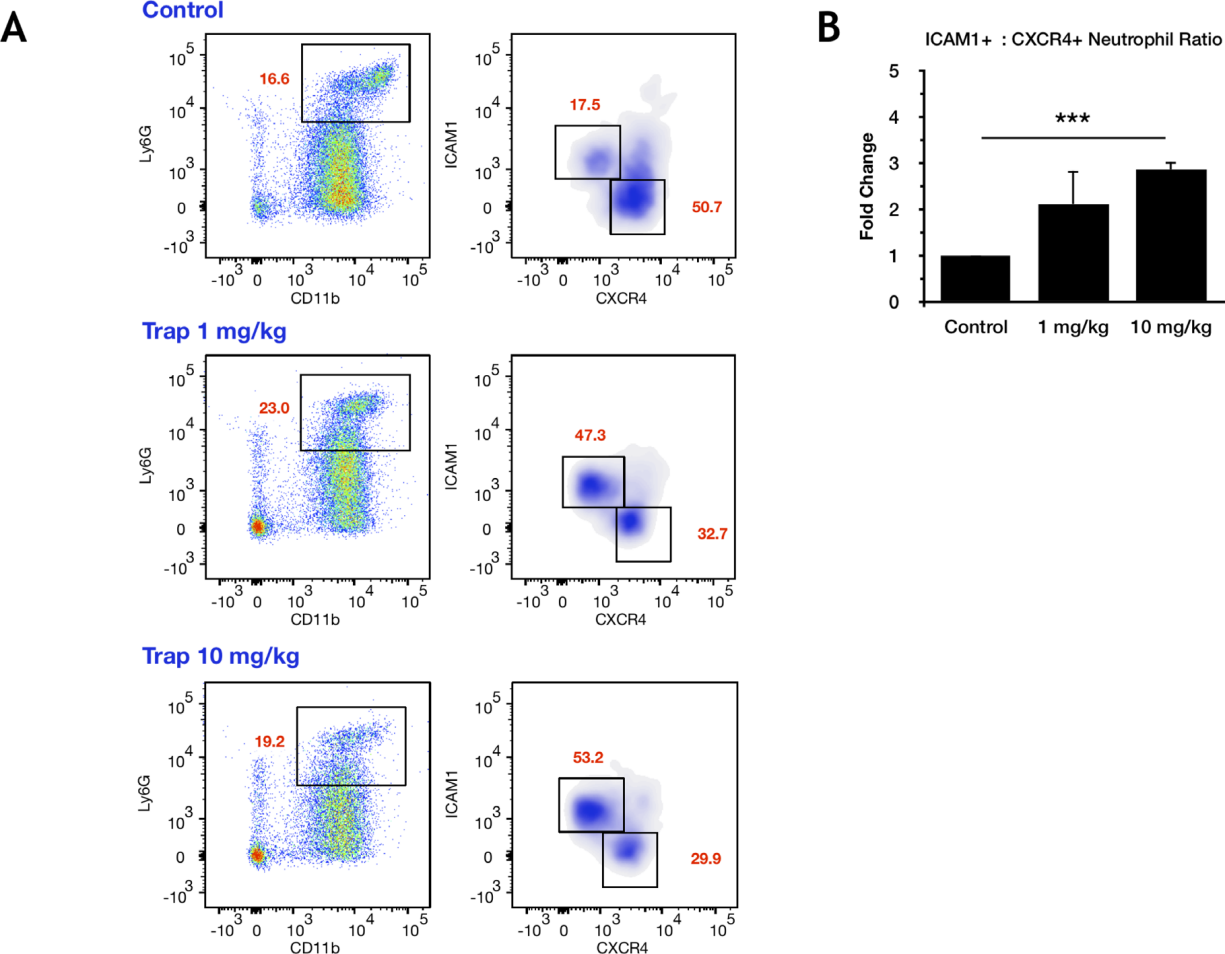
Treatment with the IGF-TRAP reduces the proportion of N2-polarized tumor-infiltrating neutrophils *in vivo* C57Bl/6 mice were injected with 2.5 × 10^5^ MC-38 cells via the intrasplenic/portal route. Treatment with 1 or 10 mg/kg IGF-TRAP was initiated one day later and continued on alternate days for a total of 3 injections per mouse. Animals were euthanized and immune cells collected 6 days post tumor injection. Shown in (**A**) are representative flow cytometric profiles of CD11b^+^Ly6G^High^ neutrophils obtained from treated and untreated mice (3–6 mice per treatment arm, *n* = 3). The percent ICAM-1^+^ or CXCR4^+^ neutrophils are indicated in red. Shown in (**B**) are the means of fold change in the ratios of ICAM-1^+^:CXCR4^+^ neutrophils relative to control, non-treated mice (±SE) that were assigned a value of 1, based on the 3 experiments. ^***^p < 0.001.

Finally, when mice inoculated with MC-38 cells were treated with 1 or 10 mg/kg of the IGF-TRAP-an inhibitor of IGF-IR signaling- [26] and their neutrophils isolated for analysis 6 days later, we found a significant and dose dependent increase in their ICAM-1:CXCR4 ratios, as assessed by flow cytometry (Figure 6A, 6B), indicating that the phenotype of TAN can be altered *in vivo* by targeting the IGF signaling axis.

## DISCUSSION

IGF-I can act directly on tumor cells to regulate their proliferation, survival, invasion, and metastasis (reviewed in [12, 27]). In addition, the IGF-axis can also affect tumor growth indirectly by modulating the host immune response (reviewed in [28]), rendering the microenvironment more, or less, permissive to cancer growth. IGF-IR targeting for cancer treatment has entered the clinical realm [26, 29–34]. An understanding of the multifaceted role that the IGF-axis plays in cancer development is important for the design of IGF-targeting regimens and the evaluation of their impact. Here, we used mice with an inducible liver IGF-I deficiency to elucidate the role of IGF-I in experimental liver metastasis of colon and lung carcinoma cells, in particular its contribution to the host innate immune response engendered by the metastatic tumor cells.

Our data show that in a microenvironment subjected to a sustained reduction in circulating IGF-I levels, neutrophil polarization to a tumor-promoting (N2) phenotype was diminished and this was associated with a decrease in liver metastases formation in 2 different tumor models of metastasis. Taken together, our results identify the IGF-I receptor as a promoter of neutrophil polarization and thereby, a contributor to the pro-metastatic microenvironment of the liver.

Several studies have examined the role of IGF-I in the regulation of neutrophil function, with seemingly conflicting results. While Himpe *et al.* identified IGF-I as a survival factor for neutrophils, delaying their Fas -mediated apoptosis through the PI3K pathway [35], Zhao *et al.* have shown that IGF-I can inhibit neutrophil activation [36]. Pretreatment of neutrophils with IGF-I was also shown to suppress phagocytosis, but this function could be restored when they were co-stimulated with PMA or E. coli [37]. These studies identified IGF-IR as a player in the regulation of neutrophil function and suggested that the role it plays can vary, depending on the inflammatory context, assay conditions and the specific function analyzed.

The function of neutrophils in the context of tumor progression is complex, as they can exert both tumor promoting and tumor inhibitory effects. Our data suggest that within the unique microenvironment of the liver, neutrophils can enhance tumor expansion by releasing tumor-promoting molecules such as VEGF, thereby, potentially, contributing to neovascularization. Our data also implicate IGF in the regulation of this tumor-promoting function. The differences we have observed in the effects of short and longer-term IGF-I deficiencies on neutrophil functions and liver metastasis are consistent with our previous observations [22]. They are most likely related to the reduction in IGF-IR expression levels, under conditions of a more sustained IGF-I deficiency. Similar findings were reported in a study of Ewing’s and osteogenic sarcoma xenografts following the exposure of the animals to an anti-IGF-IR antibody (AMG-479) for 3 weeks [38]. Recently, we documented a similar effect on hepatic stellate cells [23] that can also promote liver colonization by metastatic tumor cells. In both of these studies, reduced IGF-IR expression was observed on non-malignant cells exposed to lowered IGF-I levels for 3 weeks (but not for 2 days). Collectively the results suggest that under normal physiological conditions, IGF-I bioavailability may regulate IGF-IR expression levels on these cells. They also highlight the important role that IGF-I plays in regulating a pro-metastatic microenvironment in the liver. It should be noted however, that reciprocal communication between hepatic stellate cells and neutrophils has been documented under various inflammatory conditions [39–41]. It is therefore possible that the change in neutrophil polarization observed in iLID^3W^ mice was due, at least partially, to the reduced hepatic stellate cell activation in these mice or conversely, that reduced neutrophil polarization contributed to decreased stellate cell activation.

Neutrophil and macrophage polarization were previously shown to be driven by TGFβ [3, 6]. We have shown here that IGF-I could directly induce expression of N2-assocaited transcripts in neutrophils *in vitro* and also increased the expression of TGFβ in these cells. It is possible therefore that *in vivo,* IGF-I plays a dual role in N2 polarization by directly activating transcription of N2-associated genes and, in addition, upregulating TGFβ production, thereby indirectly contributing to an autocrine activation loop.

We observed an increase in CXCL-1 expression in whole livers of ILID^3W^ mice within 3 days post tumor injection. CXCL-1 is a major neutrophil chemoattractant [42]. This may account for the observed increase in the accumulation of Ly6G^+^ cells around tumor micrometastases, 6 days post tumor injection. The failure of the recruited neutrophils to polarize could have, in turn, decreased the incidence of metastases because N1 neutrophils are tumoricidal [6]. The source of increased CXCL-1 production in iLID mice remains to be identified, but several cell types are known to produce this chemokine, including activated endothelial cells, macrophages, neutrophils and hepatic stellate cells [39, 43, 44]. Our results suggest that IGF-I may suppress CXCL-1 production in one or more of these cells or indirectly regulate CXCL-1 levels by suppressing the expression of upstream mediators. This is in line with other studies that identified IGF-I as a mediator of immunosuppression and in particular, an inhibitor of interferon-γ [45] - a known inducer of CXCL-1[46].

Recent studies identified TAN as prognostic factors in several types of cancers, including colon carcinoma [47]. High neutrophil to lymphocyte ratios were also shown to predict a poor outcome following hepatic resection for CRC liver metastases [48]. This suggests that neutrophils infiltrating hepatic metastases may accelerate their growth. When viewed in this context, our results suggest that in addition to its direct effects on tumor cells [12], IGF-I can also promote tumor growth through its immunomodulatory effects on tumor-infiltrating neutrophils, thereby contributing to a pro-metastatic microenvironment in the liver and amplifying its tumor-growth promoting effect. Tumor- associated neutrophils have been documented at the primary and metastatic sites of different malignancies and their phenotypes could have profound effects on the type of adaptive immune response mounted [49–51]. Our findings may therefore have implications beyond the management of liver metastatic disease. Collectively, our results provide a rationale for the use of IGF-targeting strategies for the reprogramming of innate immune response cells from a tumor-promoting to a tumor-inhibiting phenotype, thereby enhancing their anti-cancer activity.

## MATERIALS AND METHODS

### Cells

The origin, properties and maintenance of murine colon adenocarcinoma MC-38 cells and Lewis lung carcinoma H-59 cells were previously described [52, 53]. Both cell lines were derived from tumors that arose in C57Bl/6 mice and were used here in the syngeneic strain. MC-38 cells were originally from an NCI repository and were obtained as a kind gift from Dr. Shoshana Yakar (New York University, NY) in 2005. They were recently authenticated by Didion and colleagues using SNP profiling, as described [54]. H-59 is a subline of the Lewis lung carcinoma that was developed by our laboratory in 1986, as described in detail previously [53]. These cells were maintained as a frozen stock and cultured for no longer than 4 weeks in DMEM (MC-38) or RPMI (H-59) media supplemented with 10% FBS and antibiotics, before the analyses described, in order to ensure the stability of their metastatic phenotypes. The cell lines were routinely tested for mouse pathogens and mycoplasma, as per the McGill University Animal Care committee guidelines and were last tested in 2016.

### Animals

All mouse experiments were carried out in strict accordance with the recommendations of the Canadian Council on Animal Care (CCAC) ‘‘Guide to the Care and Use of Experimental Animals’’ and under the conditions and procedures approved by the Animal Care Committee of McGill University (AUP # 5260). C57Bl/6 female mice were obtained from Charles River Laboratories (St. Constant, QC, Canada) and used for the experiments at the age of 7–10 wks. A colony of mice with an inducible, liver specific IGF-I deficiency (iLID) was maintained in the animal facility of the Research Institute of the McGill University Health Center, as per the guidelines of the McGill University Animal Care Committee. Newborn mice were routinely genotyped, as described in detail previously [23] and only female mice were used in this study. In these mice, a single TX injection results in gene recombination by activating the Cre recombinase gene under the control of a liver specific anti-trypsin 1α promoter. The inducible *igf1* deletion results in a 50∼80% reduction in serum IGF-I levels within ∼18 hr of a single i.p. injection of 0.3 mg TX per mouse (Supplementary Figure 3). We routinely confirmed reduced IGF-I plasma levels in all iLID mice prior to use in the experiments described (see Supplementary Table 1). Serum IGF-I levels were measure using the DuoSet IGF-I ELISA kit (R&D) that quantifies unbound IGF-I levels.

### Antibodies and reagents

The antibodies and reagents used are listed in Supplementary Table 2.

### RNA extraction and qRT-PCR

Total cellular RNA was extracted using Trizol (Life Technologies, Burlington, ON, Canada). RT-PCR was performed using the Moloney murine leukemia virus (MMLV) reverse transcriptase and Taq DNA polymerase (both from Invitrogen, Carlsbad, CA). qPCR was performed with the iQ^™^ SYBR^®^ Green Supermix (Bio-Rad Laboratories, Mississauga, ON, Canada) in a standard PCR mixture containing 0.5 µM of each primer (all primers are listed in Supplementary Table 3), 3 mM MgCl_2_, and 2 µg cDNA. Amplification and detection were performed in a BioRad LightCycler instrument (Bio-Rad Laboratories) using 25 µl reaction mixture and 40 cycles of denaturation (95° C, 10 sec) and annealing (60° C, 30 sec). A single fluorescence reading was taken at each extension step and the iQ5 software (Bio-Rad Laboratories) was used for data analysis.

### Protein extraction and Western blotting

Total cell lysates were prepared as described in detail elsewhere [55]. Serum-free neutrophil conditioned media were collected and concentrated 20-fold using Amicon Ultra 3K Centrifugal devices (EMD Millipore, Etobicoke, Ontario, Canada). Cell lysate and conditioned media proteins were separated by polyacrylamide gel electrophoresis (PAGE) using 10% SDS gels, and Western blotting performed as we previously described [55]. Primary and secondary antibody dilutions are indicated in Supplementary Table 2. The Amersham ECL Select Western Blotting Detection Reagent (GE Healthcare, Mississauga, ON, Canada) was used to detect the chemiluminescent signal.

### ELISA

The Ray Bio^©^ mouse Rantes (CCL5) ELISA kit (Ray Biotech inc, Norcross, GA) was used to measure levels of CCL5 in concentrated conditioned media and the DuoSet IGF-I ELISA kit (R&D) for measuring serum IGF-I levels, as instructed by the manufacturer.

### Experimental metastasis assays

Experimental liver metastases were generated by intrasplenic/portal injections of 2.5 × 10^4^ MC-38 cells or 10^5^ H-59 cells followed by splenectomy, as previously described [56, 57]. The number of tumor cells to be injected was determined based on preliminary dose-response analyses and selected to produce a quantifiable number of visible hepatic metastases within 14 (H-59) and 16 (MC-38) days post-tumor inoculation, at which time the animals were euthanized. Visible metastases on the surfaces of the livers were enumerated immediately after liver resection and prior to fixation.

### IHC and confocal microscopy

Cryostat sections (10 μm) were prepared from snap frozen liver fragments that were perfused with, and then fixed in a 4% paraformaldehyde solution. The sections were incubated overnight with primary antibodies diluted as indicated (Supplementary Table 2) in blocking solution (1% BSA and 1% FBS in PBS) and then for 1 hour at RT with Alexa Fluor 647 goat anti-rabbit or Alexa Fluor 568 goat anti-rat antibodies (Molecular Probes, Invitrogen), as appropriate, diluted 1:200 and used in the presence of DAPI (1:2,000). The sections were washed and mounted with the GOLD anti-fade reagent (Invitrogen) and images analyzed with a Zeiss LSM 510 Meta or a Zeiss LSM 780, confocal microscope (Carl Zeiss Canada Ltd, Toronto, Ontario, Canada) equipped with a Zen image analysis station.

### Isolation and flow cytometry of hepatic immune cells and bone marrow neutrophils

Hepatic immune cells were extracted using the protocol described in detail elsewhere [58]. Briefly, liver homogenates were prepared in cold PBS and filtered through a stainless-steel mesh using a plunger. The filtrate was centrifuged at 60 g to separate the hepatocytes, the supernatant containing the non-parenchymal fraction centrifuged at 480 g at RT and the pellet resuspended in 10 ml of a 37.5% Percoll solution in HBSS containing 100 U/ml heparin and centrifuged at 850 g for 30 min at RT to obtain an immune cell-rich fraction. Bone marrow neutrophils were isolated as described in detail elsewhere [59]. Briefly, femurs of C57BL/6 female mice were collected, and the bone marrow flushed out with a HBSS prep (HBSS with 0.5 % FBS and 20 mM HEPES), using a 20 ml syringe and a 22 G needle. The pellet was broken up by pipetting, using a 20 ml syringe and an 18 G needle. Following centrifugation, the pellet containing the bone marrow was resuspended with 5 ml HBSS prep and the cells layered on a 62% percoll solution and centrifuged to separate the neutrophils (in the pellet) from the interphase containing lymphocytes. For both procedures, neutrophils from 5–10 mice per experimental group were pooled in order to obtain adequate numbers for the analyses. Red blood cells were removed using the ACK lysing buffer prior to cell immunostaining with fluorescent-conjugated antibodies to the indicated cell surface markers and flow cytometry. Data acquisition was with a BD Canto flow cytometer and the FACsDiva software (San Jose, CA, USA) and data analysis with the FlowJo software (Ashland, OR, USA).

### Arginase assay

Arginase activity was measured in cell lysates, using the Quantichrome Arginase Assay Kit (BioAssay Systems, Hayward, CA). CD11b^+^Ly6G^+^ cells were washed with PBS and centrifuged at 4° C for 10 minutes at 1,000 g. The pellets were lysed in a 10 mM Tris-HCl (pH 7.4) buffer containing 0.4% Triton X-100, 1 mM pepstatin A, and 1 mM leupeptin and the cell lysates centrifuged at 4° C for 10 minutes at 14,000 g prior to measuring arginase activity, as per the manufacturer’s instructions.

### Nitric oxide assay

Nitric oxide activity in the conditioned media was measured using the Griess reagent system (Promega Corporations, Madison, WI). Briefly, 10^6^ CD11b^+^Ly6G^+^ cells derived from iLID^3W^ and control mice were plated in a 96 well-plate and incubated overnight at 37° C. Conditioned media were then collected, cleared of cells by centrifugation at 1000 g for 5 min and analyzed for nitrite concentration as per the manufacturer’s instructions.

### IGF-TRAP treatment

C57Bl/6 female mice were injected i.v. with 1 or 10 mg/kg of the IGF-TRAP [26] (or saline for control) 1, 3 and 5 days following the intrasplenic/portal inoculation of 2.5 × 10^5^ MC-38 cells. Livers were processed for RNA extraction and flow cytometry, 6 days post tumor inoculation.

### Statistical analysis

All cell based data (*in vitro* assays and IHC) were analyzed by a one-tailed Student’s *t*-test. The non-parametric Mann-Whitney test was used to analyze experimental metastasis data.

## SUPPLEMENTARY MATERIALS FIGURES AND TABLES


