# Supplementary material for: Loss of neutrophil polarization in colon carcinoma liver metastases of mice with an inducible, liver-specific IGF-I deficiency
Source: Oncotarget. 2018 Feb 28;9(21):15691–704. doi: 10.18632/oncotarget.24593 (PMC5884657; doi:10.18632/oncotarget.24593)
Supplement: Supplementary file 1 [file oncotarget-09-15691-s001.pdf]

Loss of neutrophil polarization in colon carcinoma liver metastases of mice with an inducible, liver-specific IGF-I deficiency

SUPPLEMENTARY MATERIALS

Supplementary Table 1: Representative serum IGF-I concentrations in ILID^{3W} and ILID^{2D} mice

Serum IGF-I concentrations (ng/ml)				
**Mouse #**	**Day 0 (pre-injection)**	**Injection**	**3 days post injection**	**3 weeks post injection**
1	405	TX	62	
2	402	TX	33	
3	399	TX	79	
4	524	TX	85	
5	342	TX	26	
6	505	TX	118	
7	326	TX	79	
8	313	OIL	359	
9	333	OIL	319	
10	345	OIL	293	
11	455	OIL	303	
12	942	TX		297
13	877	TX		234
14	470	TX		108
15	510	TX		63

Supplementary Table 2: List of all reagents and antibodies used

1) Antibodies used for IHC:
- Rabbit polyclonal antibody to cleaved caspase 3 (1:100) from Abcam (Cambridge, MA).
- Rat monoclonal antibody to CD31 (Clone MEC 13.3) (1:50) from BD Biosciences (San Jose, CA).
- Rabbit polyclonal antibody to the phosphorylated form of IGF-IR (p-IGF-IR) (Ab39398) from abcam.
- Rabbit monoclonal antibody to CXCR4 (clone UMB2) from abcam.
- Rabbit monoclonal antibody to neutrophil elastase (clone NP57) from abcam.
- Rabbit GFP-tag antibody, Alexa Fluor 488 conjugate (1:100) from Life technologies (Burlington, ON, Canada).
- Alexa Fluor 647 goat anti-rabbit and 568 goat anti-rat antibodies (both 1:200) from Molecular Probes (Eugene, OR).
- DAPI stain (1:2000) from Life technologies.

2) Antibodies used for Flow Cytometry:
- Rat anti-mouse PE-CD11b, PE-Cy7-Ly6G and APC-Ly6C antibodies (all 1:100) from Biolegend (San Diego, CA).
- BV421™ anti-mouse CD184 (CXCR4) Antibody (Clone L276F12) from Biolegend (1:200).
- Pacific Blue™ anti-mouse CD54 Antibody (Clone YN1/1.7.4) from Biolegend (1:200).
- Fixable Viability Dye eFluor® 780 (1:1000) from e-biosciences (San Diego, CA).
- Rabbit polyclonal anti-mouse total IGF-IR (1:100) from Abcam.

3) Antibodies used in Western Blotting:
- Rabbit anti-mouse IGF-IR (phospho Y1161) antibody (1:100) from abcam.
- Rabbit anti-mouse IGF-IR-β (H-60) (1:100) from Santa-Cruz (Dallas, Texas).
- HRP-conjugated goat anti-rabbit antibody (1:5000) from Jackson ImmunoResearch (West Grove, PA).

Supplementary Table 3: List of primer sets

Gene	Forward primer (5′ → 3′)	Reverse primer (5′ → 3′)
**CCL5**	GCTCCAATCTTGCAGTCG	GTCCGTGTGGGAGTAGGG
**CXCL1**	ACCCAAACCGAAGTCATAGC	TGGGGACACCTTTTAGCATC
**CXCL2**	AGTGAACTGCGCTGTCAATG	TTCAGGGTCAAGGCAAACTT
**E-selectin**	AGCTACCCATGGAACACGAC	CGCAAGTTCTCCAGCTGTT
**GAPDH**	TGTGTCCGTCGTGGATCTGA	TTGCTGTTGAAGTCGCAGGAG
**ICAM-1**	TTCACACTGAATGCCAGCTC	GTCTGCTGAGACCCCTCTTG
**IGF-IR**	GTGTGTGTCCTGGATTTGGG	GGCAGAAATGCGGAGTGGAA
**IL-1β**	GGAGAACCAAGCAACGACAAAATA	TGGGGAACTCTGCAGACTCAAAC
**IL-18**	GCCTCAAACCTTCCAAATCA	TGGATCCATTTCCTCAAAGG
**P-selectin**	TCCAGGAAGCTCTGACGTACTTG	GCAGCGTTAGTGAAGACTCCGTAT
**TGF-β1**	GACTCTCCACCTGCAAGACC	GGACTGGCGAGCCTTAGTTT
**VCAM-1**	GTGAAGATGGTCGCGGTCTT	GGCCATGGAGTCACCGATT
**VEGF**	GGAGTCTGTGCTCTGGGATT	AGAACCAACCTCCTCAAACCG

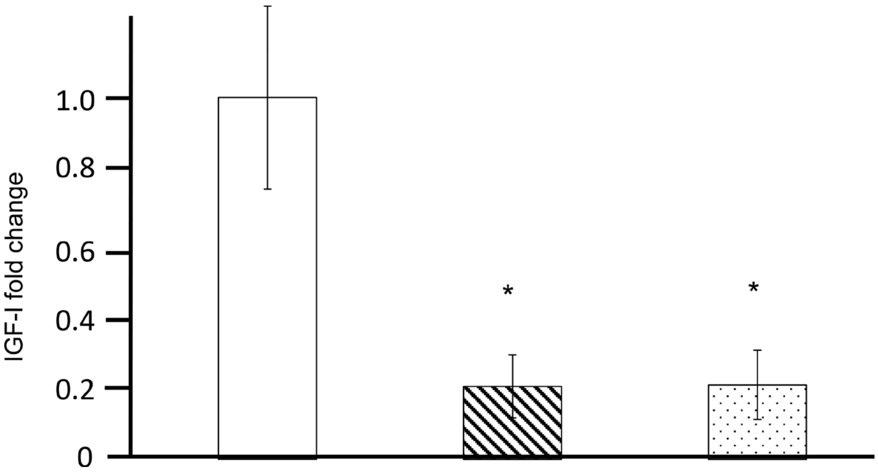

Supplementary Figure 1: Serum IGF-I levels are similarly decreased in iLID^{2D} and iLID^{3W} mice. Serum was collected from iLID mice prior to TX injection (open bar) and 2 days (dashed bar) or 3 weeks (dotted bar) post TX injection. IGF-I levels were measured using a mouse/rat Duoset IGF-I ELISA kit as per manufacturer's instructions. Note that the DuoSet ELISA kit measures free, unbound IGF-I only. For each mouse, triplicate samples were analyzed. Results are means (± SE) based on 7 mice per group. $^{*}p < 0.05$.

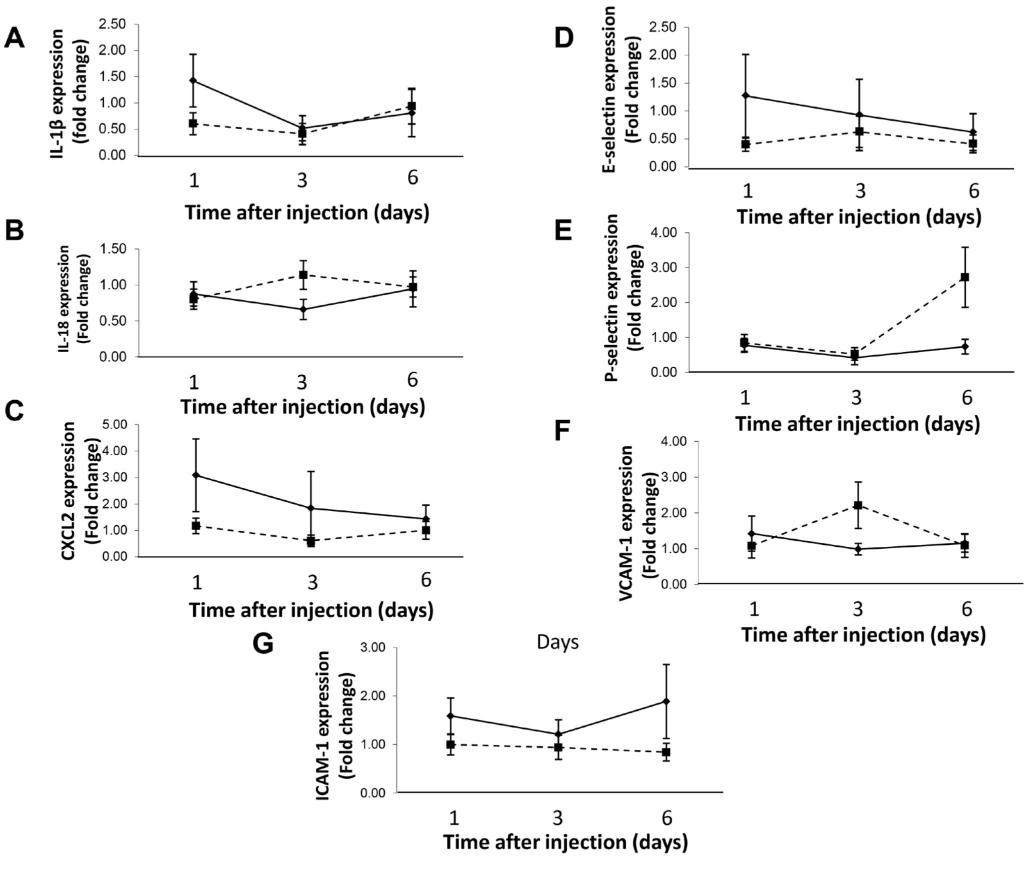

Supplementary Figure 2: The effect of a sustained IGF-I deficiency on the expression of liver inflammatory mediators is selective. iLID mice were injected i.p. with tamoxifen or sunflower seed oil (vehicle), 3 weeks prior to the injection of 2.5×10^5 MC-38-GFP cells via the intrasplenic/porta route. Mice were sacrificed 1, 3 or 6 days following tumor cell injection and a liver fragment collected from each mouse (3 mice per group) and used for RNA extraction and analysis by qPCR. Shown are mean expression levels (± SE) for the indicated transcripts in tamoxifen-treated (solid lines) and vehicle-treated (dashed lines) mice normalized to GAPDH ($p > 0.05$ for all transcripts shown). For each panel (**A**–**G**), the inflammatory mediator analyzed is identified on the Y-axis descriptor.

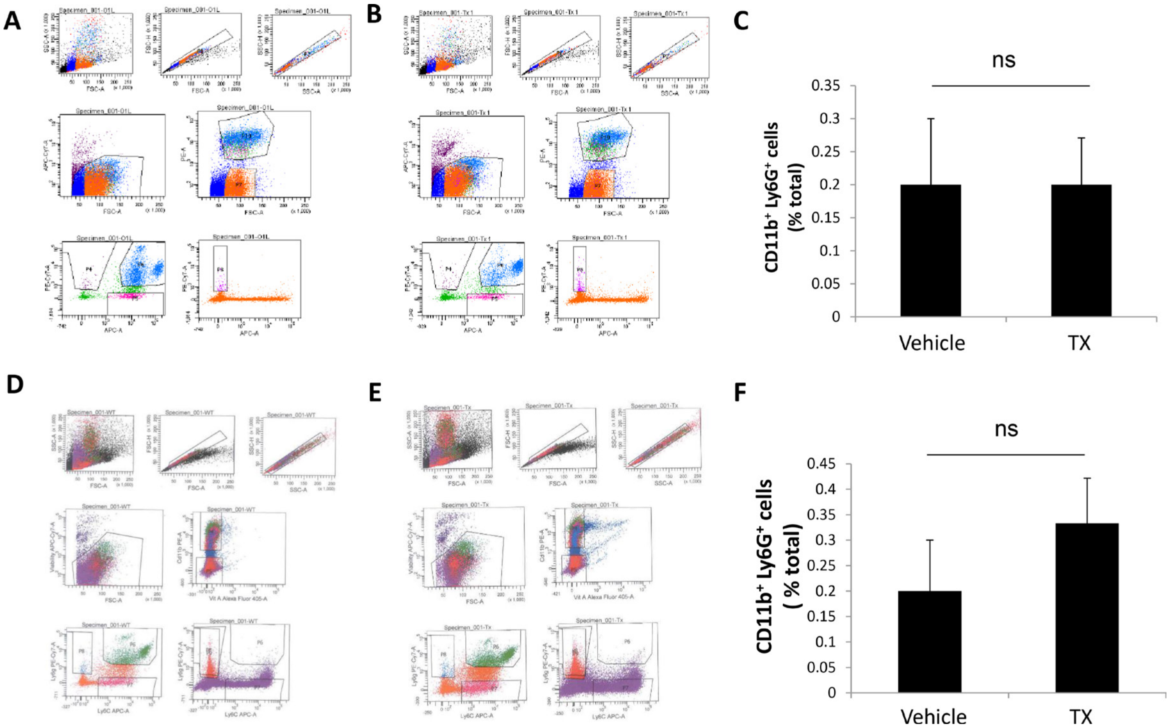

Supplementary Figure 3: A sustained IGF-I deficiency does not alter the total number of CD11b^{+} Ly6G^{+} cells in the liver. iLID mice were injected i.p. with TX or sunflower seed oil (vehicle) 2 days (**A**–**C**) or 3 weeks (**D**–**F**) prior to the injection of 2.5 × 10^{5} MC-38-GFP cells via the intrasplenic/portal route. Mice were sacrificed 6 days later, hepatic immune cells isolated and CD11b^{+}Ly6G^{+} cells analyzed by flow cytometry. Shown in (A, B, D, E) are representative flow cytometry profiles and in (C, F) mean numbers of CD11b^{+}Ly6G^{+} cells based on 3 livers per group and expressed as percent of total immune cells (±SE).

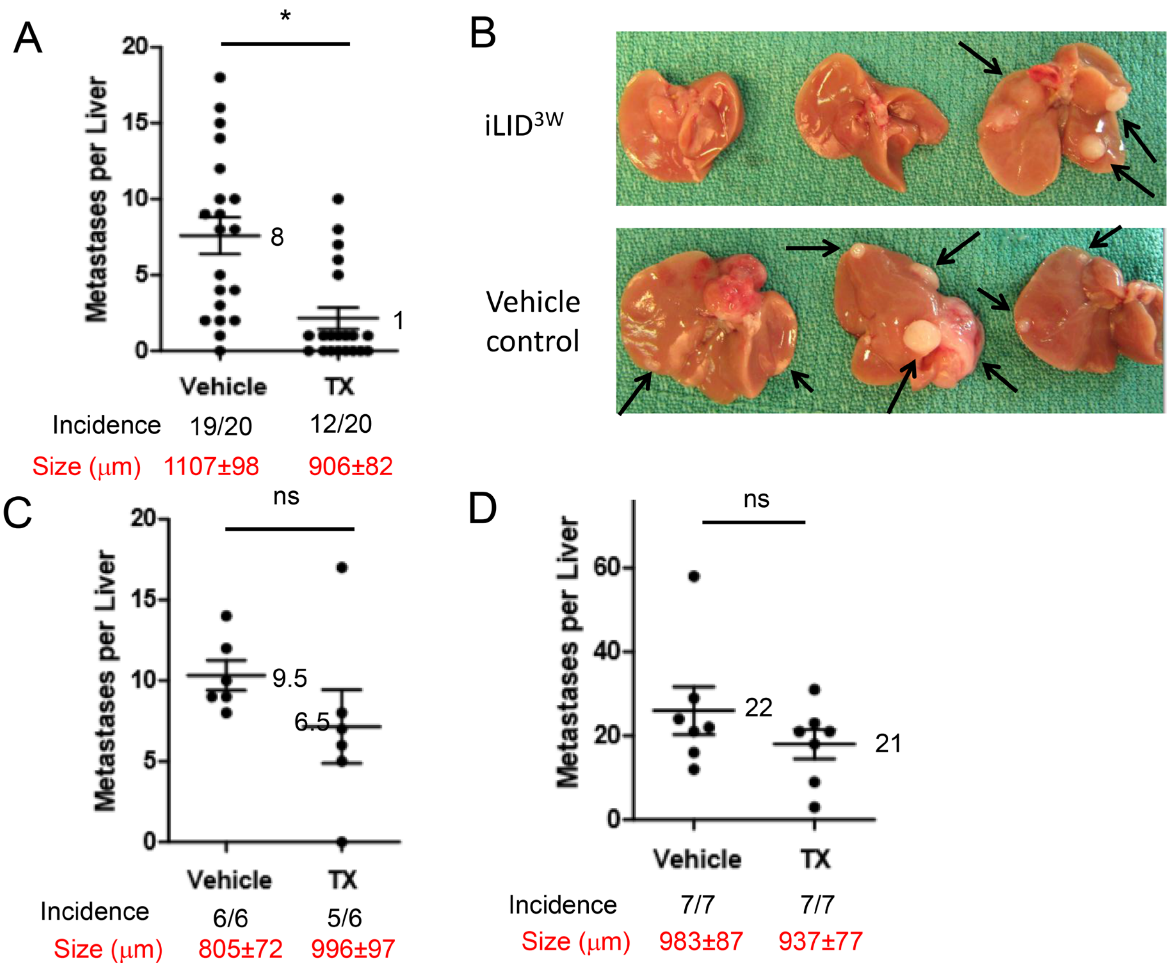

Supplementary Figure 4: A sustained liver IGF-I deficiency decreases the number of experimental liver metastases. iLID mice received a single i.p. injection of 0.3 mg TX or sunflower seed oil (vehicle) 3 weeks (**A**, **B**) or 2 days (**C**) prior to the injection of 2.5×10^4 MC-38 cells via the intrasplenic/portal route. Age matched C57BL/6 female mice injected (or not) with 0.3 mg TX i.p. were used as a second control group and were injected with 5×10^4 MC-38 cells (**D**). Mice were sacrificed 16 days post tumor cell injection and metastases visible on the surfaces of the livers enumerated prior to fixation. Shown in (A, C, D) are the numbers of metastases seen on the individual livers in each group. Bars denote medians. The numbers of mice in each group that developed hepatic metastases are indicated on the bottom of each column. The average sizes of the metastases (± SD) are shown below each column. Shown in (B) are representative livers from one of 3 experiments. ns = not significant, $^*p < 0.05$ as determined by the Mann–Whitney non-parametric test.
